# A Haplotype Associated with Enhanced Mineralocorticoid Receptor Expression Facilitates the Stress-Induced Shift from “Cognitive” to “Habit” Learning

**DOI:** 10.1523/ENEURO.0359-17.2017

**Published:** 2017-11-15

**Authors:** Lisa Wirz, Martin Reuter, Jan Wacker, Andrea Felten, Lars Schwabe

**Affiliations:** 1 Department of Cognitive Psychology, University of Hamburg, Hamburg 20146, Germany; 2 Department of Differential and Biological Psychology, University of Bonn, Bonn 53111, Germany; 3 Department of Differential Psychology, University of Hamburg, Hamburg 20146, Germany

**Keywords:** glucocorticoids, hippocampus, mineralocorticoid receptor, stress

## Abstract

Stress induces a shift from hippocampus-dependent “cognitive” toward dorsal striatum-dependent “habit” memory. However, not all individuals are susceptible to this shift under stress. Based on pharmacological studies indicating a critical role of the mineralocorticoid receptor (MR) in the stress-induced bias toward dorsal striatal learning, we hypothesized that *MR* gene variants contribute to these individual differences. In two experiments, healthy participants were genotyped, exposed to a stressor or control manipulation and performed a learning task that can be solved using hippocampal or dorsal striatal systems, while electroencephalography (EEG; Experiment I) or functional magnetic resonance imaging (fMRI; Experiment II) measurements were taken. Stress led to a shift from hippocampal to dorsal striatal learning which was more pronounced in homo- and heterozygous carriers of a six single nucleotide polymorphisms (SNPs)-comprising haplotype containing the alleles of two *MR SNPs* associated with increased MR expression and transactivational activity (*MR*-2G/C **C** [rs2070951], *MR*-I180V **A** [rs5522]). This stress-induced shift toward habit memory was paralleled by an increased feedback-related negativity (FRN), which may reflect striatal processing, and increased caudate activation. Carriers of the *MR* haplotype showed a reduced P3a, an event-related potential thought to indicate cognitive processing, and reduced hippocampal activity after stress. Moreover, stress resulted in reduced amygdala-hippocampus connectivity and the decrease in amygdala connectivity to the parahippocampal cortex was particularly pronounced in *MR* haplotype carriers. Our findings indicate that genetic variants associated with enhanced MR expression facilitate a stress-induced shift from hippocampal toward dorsal striatal learning, most likely via impaired hippocampal processing and reduced amygdala-hippocampus cross talk, allowing the dorsal striatum to guide behavior under stress.

## Significance Statement

Stressful events may trigger a shift from hippocampus-dependent, “cognitive” toward dorsal striatum-dependent, “habitual” control of learning. While being generally adaptive for performance under stress, this shift may contribute to stress-related psychopathology. However, there are substantial individual differences in the stress-induced bias toward habit learning, the source of which is not fully understood. In line with pharmacological studies pointing to a critical role of the mineralocorticoid receptor (MR) in the stress-induced learning bias, we report here that a *MR* haplotype associated with enhanced MR expression facilitates habit learning under stress. Using electroencephalography (EEG) and functional magnetic resonance imaging (fMRI), we show that this genetic modulation is most likely mediated by altered hippocampus activity and amygdala-hippocampus crosstalk.

## Introduction

Stressful events may modulate the engagement of multiple, anatomically and functionally distinct memory systems (Packard and Wingard, 2004; Schwabe, 2013; Schwabe and Wolf, 2013). Specifically, stress has been shown to favor simple but rigid “habit” learning supported by the dorsal striatum over more complex “cognitive” learning dependent on the hippocampus or prefrontal cortex (Kim et al., 2001; Schwabe et al., 2007; Schwabe and Wolf, 2009, 2012). This stress-induced bias toward habit learning is thought to contribute to stress-related psychopathologies, including posttraumatic stress disorder (PTSD) and addiction (Schwabe et al., 2010a, 2011; Packard and Goodman, 2012).

The shift from cognitive to habit learning under stress may be accompanied by reduced hippocampal and increased dorsal striatal activity and evidence suggests that the amygdala orchestrates the engagement of these memory systems (Packard and Wingard, 2004; Schwabe and Wolf, 2012; Schwabe et al., 2013; Vogel et al., 2015). These stress-induced changes in the preferential engagement of multiple memory systems are critically driven by glucocorticoids (mainly cortisol in humans) binding to membrane-bound mineralocorticoid receptors (MRs; Vogel et al., 2016). In particular, pharmacological studies in rodents and humans showed that blockade or absence of MRs prevented the stress-induced bias toward dorsal striatum-dependent memory (Schwabe et al., 2010b; ter Horst et al., 2012; Schwabe et al., 2013).

However, not all individuals show the bias toward dorsal striatal habit learning under stress. Given the potential clinical relevance of the stress-induced memory bias, it is important to identify factors that contribute to this individual variance. If the stress-induced shift toward habit memory is mediated by MRs, genetic differences in the *MR* gene (*NR3C2*) are a likely source contributing to individual differences in the engagement of multiple memory systems under stress. Two common variants in the *MR* gene, the *MR*-2G/C **C** (rs2070951) and *MR*-I180V **A** (rs5522) alleles, are associated with increased expression and transactivation capacity of the MR *in vitro* (DeRijk and De Kloet, 2008; van Leeuwen et al., 2010) and altered hypothalamic-pituitary-adrenal (HPA) axis reactivity (DeRijk, 2009). Similarly to pharmacological blockade of the MR, *MR*-I180V **G** and *MR*-2G/C **G** allele carriers showed increased levels of cortisol in response to psychosocial stress (DeRijk et al., 2006). In addition, these *MR* single nucleotide polymorphisms (SNPs) together result in four haplotypes (GA, CA, CG, GG).The common and functional CA haplotype, which results in higher transcriptional, translational and transactivational *MR* activity, has been associated with enhanced resilience to depression (Klok et al., 2011) as well as traumatic stress (ter Heegde et al., 2015; de Kloet et al., 2016).

The present study aimed to test whether *MR* haplotypes with known differences in *MR* transactivation and expression contribute to individual variance in stress effects on multiple memory systems. For this purpose we conducted two independent experiments in which healthy participants, genotyped for several *MR* haplotypes, were exposed to a stressor (or control manipulation) before completing a probabilistic classification learning (PCL) task that can be solved using hippocampus-dependent single-cue or dorsal striatum-dependent multi-cue strategies (Gluck et al., 2002; Shohamy et al., 2004; Schwabe and Wolf, 2012). In Experiment I, we used electroencephalography (EEG) to assess the feedback-related negativity (FRN) and the P3, event-related brain potentials (ERPs) reflecting, at least partly, dorsal striatal (Nieuwenhuis et al., 2005; Hauser et al., 2014) and hippocampal processes (Knight, 1996; Polich, 2007), respectively. In Experiment II, we employed functional magnetic resonance imaging (fMRI) to elucidate the neural underpinnings of modulatory effects of the *MR* haplotype on the stress-induced shift toward habit memory. We hypothesized that the *MR* haplotype associated with increased MR functionality (*MR*-2G/C **C** and *MR*-I180V **A**) enhances the stress-induced shift from hippocampal toward dorsal striatal memory processes. At the neural level, we expected this shift to be mediated by changes in P3 and FRN magnitude, as well as by alterations in activation of the dorsal striatum and hippocampus and in connectivity of these memory systems with the amygdala.

## Materials and Methods

### Experiment I: *MR* haplotype, stress, and the engagement of multiple memory systems

#### Participants and experimental design

Healthy volunteers (*N* = 252) without current or previous neurologic or psychiatric disorders or present medication intake participated in this experiment (127 women; mean age: 25.1 years, SD 3.5 years). Factors influencing the reactivity of the HPA axis were controlled for by excluding smokers and women taking hormonal contraceptives and by testing women outside their menstrual cycle phase (Kirschbaum et al., 1999; Rohleder and Kirschbaum, 2006). To control for the diurnal rhythm of cortisol, all testing took place in the afternoon. The experiment was approved by the ethical review board of the German Psychological Society (reference: LS072014). Participants gave written informed consent and received a moderate monetary compensation of 25€ for their participation. This sample is part of a larger project on individual differences in stress effects on multiple memory systems (Wirz et al., 2017).

To examine modulatory effects of an *MR* haplotype on stress-induced changes in the preferential engagement of multiple memory systems, we used a 2 × 2 between-subjects design with the factors treatment (stress vs control manipulation) and *MR* haplotype (homo- and heterozygous carriers vs noncarriers) in which participants were randomly assigned to the stress or control condition. Due to technical difficulties and excessive artifacts in the EEG, 24 participants were excluded from the ERP analyses, leading to a sample of 228 participants (stress: 81 carriers, 33 noncarriers; control: 91 carriers, 23 noncarriers), whereas for the behavioral analyses data from all 252 tested participants were used (stress: 90 carriers, 36 noncarriers; control: 101 carriers, 25 noncarriers).

#### Genetic analyses

Participants were genotyped for seven SNPs of the gene coding for the *MR* (*NR3C2*; rs1512344, rs2070950, **rs2070951 [*MR*-2G/C]**, rs4835519, **rs5522 [*MR*-I180V]**, rs5534, rs7658048). Two of them are functional SNPs located on exon 2 of the *MR* gene (*MR*-2G/C, allele frequency 50% and *MR*-I180V, allele frequency 12%) that may alter HPA axis responsiveness, thereby affecting individual stress responsivity and vulnerability to stress-related disorders (DeRijk, 2009; Klok et al., 2011; van Leeuwen et al., 2011; Medina et al., 2013). For genetic analysis, DNA was extracted from buccal cells. Automated purification of genomic DNA was conducted by means of the MagNA Pure LC system using a commercial extraction kit (MagNA Pure LC DNA isolation kit; Roche Diagnostics). Genotyping of the *MR* polymorphisms was performed by MALDI-TOF mass spectrometry using the iPLEX assay and the Sequenom MassARRAY platform.

Linkage analyses between SNPs and construction of haplotype blocks were conducted by means of Haploview 4.2 (https://www.broadinstitute.org/haploview/haploview). Haplotype blocks were defined by the method suggested by (Gabriel et al., 2002). Individual haplotypes were calculated with PHASE, version 2.1. PHASE implements a Bayesian statistical method for reconstructing haplotypes from population genotype data. To test for deviations from Hardy-Weinberg Equilibrium, allele frequencies were analyzed using χ^2^ tests. Homo- and heterozygous carriers of the *MR*-2G/C **C** and *MR*-I180V **A** haplotype (= *MR* haplotype carriers), showing higher *MR* transactivation and expression compared to the other haplotypes (GA, CG, GG), were treated as one group and tested against all other haplotypes (= *MR* haplotype noncarriers) in further analyses.

#### Stress and control manipulation

Participants in the stress condition underwent the Trier Social Stress Test (TSST), which is known to reliably increase activity of the autonomic nervous system (ANS) and the HPA axis (Kirschbaum et al., 1993). After 3 min of preparation, each participant was asked to give a 5-min free speech about why he or she is the ideal candidate for a job tailored to his or her interests and subsequently had to solve a difficult mental-arithmetic task for another 5 min (counting backwards from 2043 in steps of 17). Throughout the TSST, participants were videotaped and evaluated by a reserved and nonreinforcing panel. In the control condition, participants talked about a self-chosen topic and performed an easy calculation task (counting forward in steps of 15) without panel and video recordings.

To assess the effectiveness of the stress induction, subjective and physiologic measures were taken at several time points across the experiment. Changes in subjective mood were evaluated using a German mood questionnaire (MDBF; subscales: depressed vs elevated, restless vs calm, sleepy vs awake; high scores indicate elevated mood, calmness, and wakefulness; Steyer et al., 1994). Additionally, participants rated the difficulty, unpleasantness and stressfulness of the stress or control manipulation on a scale from 0 (“not at all”) to 100 (“very much”). Blood pressure was measured using a Dinamap system (Critikon) before (−25 min), during (+10 min), and after (+20 min, +60 min, +80 min) the experimental manipulation. Furthermore, saliva samples were collected before (-25 min) and after (+20 min, +30 min, +40 min, +80 min) the experimental treatment using Salivette collection devices (Sarstedt). Saliva samples were stored at −18°C until the free fraction of cortisol was determined using commercially available chemiluminescence immunoassays (IBL).

#### PCL task

To assess the engagement of multiple memory systems, participants completed a modified version of the weather prediction task (Knowlton et al., 1994; Knowlton et al., 1996), while EEG was recorded and ∼15 min after the treatment, when peak cortisol levels after stress were expected. In this PCL task, participants learned to classify stimuli into the categories “rain” and “sun” based on trial-by-trial feedback. One, two or three (out of four) cards appeared on each trial, yielding 14 different cue patterns. These cue patterns were associated with the outcomes sun and rain in a probabilistic manner, such that a particular cue was associated with the outcome sun with a probability of 75.6, 57.5, 42.5, or 24.4 percentage across 100 trials; these probabilities are in line with previous studies using this task (Gluck et al., 2002; Lagnado et al., 2006; Schwabe and Wolf, 2012; Schwabe et al., 2013). A response was counted as correct if it matched the outcome with the highest probability for that cue pattern. Participants completed 100 PCL trials (duration: ∼25 min). On each trial, 1 of the 14 cue patterns appeared and participants had 5 s to respond by pressing one of two buttons that corresponded with the outcomes sun and rain. Responses were highlighted with a red circle (500 ms) before a black screen appeared (500 ms), which was followed by a feedback stimulus in the form of a happy or sad face (1.000 ms). The intertrial interval varied between 1 and 2.5 s.

#### Assessment of learning strategies

The PCL task can be solved by using different learning strategies that rely on distinct brain systems. Patient and neuroimaging studies showed that participants may acquire the task using single-cue strategies supported by a hippocampus-dependent system or by using multi-cue strategies that are based on the dorsal striatum (Knowlton et al., 1996; Shohamy et al., 2004; Foerde et al., 2006; Schwabe and Wolf, 2012). In order to assess participants’ learning strategies during PCL, participants’ actual responses were compared with ideal response patterns for each strategy (Gluck et al., 2002; Lagnado et al., 2006). A least mean squares measure resulted in a fit value ranging from 0 to 1 (0 indicating a perfect fit). Participants were assigned the strategy with the best fit score. If none of the scores for all possible strategies was <0.16, participants’ strategies were classified as “nonidentifiable” (Gluck et al., 2002; Wirz et al., 2017). Independent of the experimental group, no strategy was identifiable in 20 participants (χ^2^_(1)_ = 0.220, *p* = 0.639). In line with previous studies (Schwabe and Wolf, 2012; Schwabe et al., 2013), strategies were classified into hippocampus-dependent single-cue and dorsal striatum-dependent multi-cue strategies. Although this dichotomization may reduce some of the variation, the classification into single- and multi-cue strategies (above the actual fit score and the substrategies) is useful to analyze differences in the predominant engagement of either one of these systems, and it promotes the comparison to previous studies using this task (Shohamy et al., 2004; Schwabe and Wolf, 2012; Schwabe et al., 2013).

#### Behavioral and physiologic data analyses

Subjective and physiologic measurements were analyzed using mixed-design ANOVAs with time as within-subject factor and treatment (TSST vs control) as well as *MR* haplotype (carriers vs noncarriers) as between-subjects factors. A mixed-design ANOVA with blocks of 10 trials as within-subject factor was used to assess learning performance on the PCL task. Group differences in learning strategy were analyzed by means of χ^2^ tests. Statistical analyses were performed using SPSS Statistics 22 (IBM). All reported *p* values are two-tailed. In case of violation of the sphericity assumption, Greenhouse-Geisser corrections were applied. Significant main and interaction effects were followed by the appropriate *post hoc* tests.

#### EEG recording and analyses

During the PCL task, EEG was recorded from 64 active electrodes arranged according to the international 10–20 system. Horizontal electro-oculograms were measured and the most frontal electrodes Fp1 and Fp2 served as recording sites for vertical eye movements. A Biosemi Active-Two amplifier system was used with a sampling rate of 2048 Hz (Biosemi). Common mode sense and driven right leg electrodes served as recording reference and ground.

EEG data were analyzed offline using the Brain Vision Analyzer software (Brain Products). After the EEG signal was downsampled to 512 Hz, the data were high-pass filtered at 0.01 Hz. To remove artifacts from electrical lines, a 50 Hz notch filter was applied. EEG data were then visually inspected to discard any extreme artifacts. Additionally, artifacts originating from eye-blinks or -movements were removed using an independent component based approach. Bad channels were replaced by means of topographic interpolation and the data were re-referenced to the average of all electrodes. To analyze ERPs reflecting feedback processing, data were segmented into epochs from −200 to 800 ms with respect to feedback stimulus onset and subsequently baseline corrected relative to the 200 ms preceding the feedback stimulus. Before averaging, trials were rejected if there was a voltage step higher than 50 µV/ms, or a difference of >100 µV as well as a signal lower than 0.1 µV was detected in any of the intervals.

The FRN, and event-related potential which likely reflects striatal feedback processing (Nieuwenhuis et al., 2005; Hauser et al., 2014), was calculated as the most negative peak amplitude in the time window between 200 and 350 ms following feedback presentation relative to the preceding positive peak amplitude between 150 ms and the latency of that negative peak (Eppinger et al., 2008; Rustemeier et al., 2013). A mixed-design ANOVA with electrode site and feedback (positive vs negative) as within-subject factors and treatment as well as *MR* haplotype as between-subjects factors was used to investigate stress- or genotype-related differences in the FRN. Feedback was added as a factor, since the FRN is particularly important for learning from negative feedback (van der Helden et al., 2010). For each participant, on average 37.5 (SD = 13.8 trials) negative feedback trials were available. Frontal electrodes (FC1, Fz, FCz, FC2), where the FRN was most pronounced, were included in the analyses.

The P3a and P3b components are proposed to reflect cognitive mechanisms facilitating attention and promoting memory processes and to involve frontal areas and the hippocampus (Knight, 1996; Polich, 2007). The P3a was calculated as the mean activity in a time window between 235 and 425 ms at central electrodes (C1, Cz, C2). The P3b was defined as the mean activity in a time window between 270 and 420 ms at parietal electrodes P1, Pz, P2. Since no differences between negative and positive feedback in P3a or P3b were expected, analyses included all feedback trials (mean number of trials = 77, SD = 17 trials). Repeated measures ANOVA with electrode site as within-subject factor and treatment as well as *MR* haplotype as between-subjects factors were used to investigate stress- or genotype-dependent differences in the P3 components.

### Experiment II: Neural signature of *MR* haplotype modulation of stress-induced changes in multiple memory systems

#### Participants and experimental design

A total of 128 volunteers of the Bonn Gene Brain Behavior Project participated in this experiment (62 women; mean age = 23.0 years, SD = 3.6 years). Participants were healthy, young nonsmokers without medication intake, or lifetime history of any neurologic or psychiatric disorders. Furthermore, any contraindications for fMRI measurements served as exclusion criteria. The experiment was approved by the ethical review board of the German Psychological Society (DGPs; reference: LS072014) as well as by the local committee at the University of Bonn. Participants gave written informed consent and received a moderate monetary compensation of 35 €. This sample is part of a larger project on individual differences in stress effects on multiple memory systems (Wirz et al., 2017).

In line with the first experiment, we used a 2 × 2 between-subjects design with the factors treatment (TSST vs control manipulation) and *MR* haplotype (carriers vs noncarriers). Participants were randomly assigned to the stress or control condition. Due to technical difficulties and excessive head motion in the MRI scanner, 8 participants were excluded from the fMRI analyses, leading to a sample of 120 participants (stress: 47 carriers, 13 noncarriers; control: 45 carriers, 15 noncarriers) for the fMRI analyses. For the behavioral analyses, data from all 128 tested participants were used (stress: 50 carriers, 15 noncarriers; control: 48 carriers, 15 noncarriers).

#### Experimental procedure

The experimental procedure, including the stress manipulation, the parameters measured and the PCL task, was identical to the first experiment, except that fMRI instead of EEG measurements were taken and that the PCL task was slightly modified to accommodate fMRI requirements. More specifically, in addition to 100 PCL trials, participants completed 100 visuomotor control trials in which they were asked to indicate whether <2 or ≥2 cards appeared on the screen (trial type was randomly alternated; task duration: ∼45 min). Additionally, the timing of the events was adjusted to the slow blood oxygenation level-dependent (BOLD) response. In Experiment II, there were 31 participants for whom no strategy could be identified; experimental groups did not differ in that number (*p* = 0.316). The behavioral analyses were in line with those of the first experiment.

#### MRI acquisition and analyses

MRI measurements were acquired using a 3T Trio Scanner (Siemens) equipped with a 32-channel head coil. BOLD T2-weighted echoplanar functional images were acquired parallel to the anterior commissure-posterior commissure plane (37 transversal slices; TR = 2000 ms; TE = 30 ms; ascending acquisition; effective voxel size = 3 × 3 × 3 mm). Additionally, a high-resolution T1-weighted anatomic image was acquired (208 sagittal slices, TR = 1660 ms, TE = 2.54 ms, voxel size = 0.8 × 0.8 × 0.8 mm).

Preprocessing and analyses of the fMRI data using general linear modeling were performed with the SPM12 Matlab toolbox (Wellcome Trust Center for Neuroimaging). Functional data were slice-time and head-motion corrected as well as coregistered to the structural image using rigid-body transformations. The T1-weighted image was segmented into gray and white matter, cerebrospinal fluid, bone, soft tissue, and air. Forward deformation fields were then used to spatially normalize the functional and structural scans to the Montreal Neurologic Institute standard brain. Finally, normalized functional images were smoothed using an 8-mm full-width half-maximum Gaussian kernel.

Correct and incorrect PCL trials as well as visuomotor control trials were modeled using canonical hemodynamic response functions. Additionally, fixation, button presses and the six movement parameters were included into the model. Data were filtered in the temporal domain using a nonlinear high-pass filter with a 128-s cutoff. Contrast images were generated for PCL minus control trials and for correct minus incorrect PCL trials. These difference contrasts were then entered into second-level (group) analyses, using a full-factorial model with the factors treatment (control vs stress) and *MR* haplotype (carriers vs noncarriers). Psycho-physiologic interaction (PPI) analyses were performed to assess whether the coupling of the amygdala with the hippocampus, the dorsal striatum and the putamen was altered by stress and/or *MR* haplotype. For this purpose, the first eigenvariate of the time course of each ROI in the contrast PCL correct minus PCL incorrect was extracted from the appropriate brain atlases and used as seed. The PPI was then computed as the element-by-element product of the BOLD signal time course of this seed and a vector coding for successful classification learning. Next, each time course was added separately as a covariate of interest in addition to the first-level regressors. The individual PPI contrasts were then entered in a second-level random-effects analysis. Results of these analyses give insight into brain regions that show a similar and task-dependent pattern of activation. These regions are therefore supposed to be functionally connected during correct classification learning.

Explorative whole brain analyses as well as ROI analyses were used. A priori ROIs were the memory system structures of interest (hippocampus, caudate nucleus and putamen) as well as the amygdala because this area is assumed to modulate multiple memory systems (Elliott and Packard, 2008; Schwabe et al., 2013; Vogel et al., 2015) and is affected by MR activation (Karst et al., 2010). Anatomic masks of the caudate nucleus, the putamen and the amygdala were taken from the Harvard-Oxford subcortical atlas, whereas masks of the hippocampal subregions were taken from the Anatomy Toolbox for SPM (Institute of Neuroscience and Medicine). For the explorative whole-brain analysis, the significance threshold was set to *p* < 0.05 at cluster level and corrected for multiple testing [familywise error (FEW) correction]. ROI analyses were performed using small-volume correction with an initial threshold of *p* < 0.05 uncorrected, followed by FEW correction (*p* < 0.05). Thresholds at 50 percentage were used to include only voxels with a probability of at least 50 percentage to belong to each subregion.

## Results

### Experiment I: *MR* haplotype, stress, and the engagement of multiple memory systems

#### MR haplotype analyses

**Figure 1.**
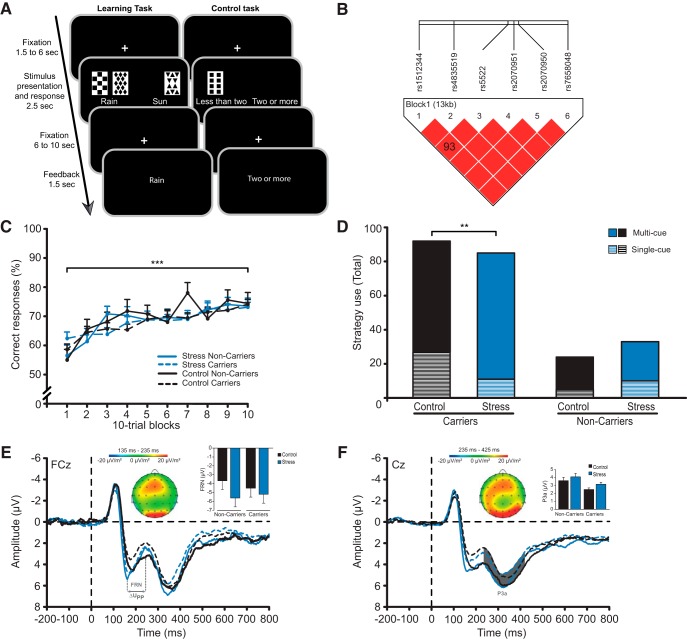
PCL task, haplotype analysis, and behavioral and EEG data of Experiment I. ***A***, Participants were required to learn how to predict the weather (rain or sun) from presentation of three to four out of four possible cues based on trial-by-trial feedback. In Experiment II, participants also completed a visual-motor control task in which they had to indicate whether ≤2 or >2 cards were presented. ***B***, Haplotype block covering 13 kb on the *MR* gene. Homo- and heterozygous *MR* haplotype carriers (*MR*-2G/C **C** [rs2070951], *MR*-I180V **A** [rs5522]) were tested against all other haplotypes. ***C***, Classification learning performance increased across trials but was unaffected by stress or the *MR* haplotype. ***D***, Stress, however, increased the use of multi-cue strategies, thought to rely on the dorsal striatum, and decreased the use of single-cue strategies, assumed to be supported by the hippocampus. This bias toward enhanced dorsal striatal processing was only observed in stressed *MR* haplotype carriers. ***E***, EEG data revealed a significant stress-induced increase in the FRN at FCz electrode, which was calculated as the most negative peak amplitude in the time window irrespective between 200 and 350 ms following feedback presentation relative to the preceding positive peak amplitude between 150 ms and the latency of that negative peak. ***F***, *MR* haplotype carriers showed, irrespective of stress, an enhanced P3a at central electrodes (C1, Cz, C2), which was calculated as the mean activity in the time window between 235 and 425 ms following feedback presentation. ΔU_PP_ represents the voltage difference between the positive and the negative peak amplitude after negative feedback. ***p < 0.001, **p < 0.01, error bars represent SEM.

Haplotype analyses revealed significantly strong linkage between six *MR* SNPs (rs1512344, rs2070950, **rs2070951**, rs4835519, **rs5522**, rs7658048) building a haplotype block covering 13 kb on the *NR3C2* gene (Fig. 1*B*). *MR* haplotype details and allele frequencies can be found in Tables 1 and 2. The *MR* haplotype, including the *MR*-2 G/C **C** and *MR*-I180V **A** alleles previously associated with increased MR expression and transactivational capacity (DeRijk and De Kloet, 2008; van Leeuwen et al., 2010), was of particular interest (alleles in order of the SNPs: CC**C**T**A**G). Experiment I included 132 participants carrying one and 59 participants carrying two alleles of this haplotype. Another four haplotypes were identified and one participant carried an unknown haplotype (Tables 1 and 2). Importantly, however, that participant did not carry the **C** and **A** alleles of the *MR* SNPs of interest (**rs2070951** and **rs5522** respectively). Homo- and heterozygous carriers of the CC**C**T**A**G haplotype (= *MR* haplotype carriers), which shows higher *MR* transactivation and expression compared to the other haplotypes, were tested against all other haplotypes (= *MR* haplotype noncarriers), leading to 90 carriers and 36 noncarriers in the stress and 101 carriers and 25 noncarriers in the control condition. Frequencies of the *MR* SNPs were in accordance with those documented in the database of the National Center for Biotechnology Information (NCBI) for Europeans. Frequencies were in Hardy-Weinberg equilibrium (all *p* ≥ 0.23) and the distribution of carriers and noncarriers of the *MR* haplotype did not differ in the stress (90 carriers, 36 noncarriers) and control group (101 carriers, 25 noncarriers; χ^2^_(1)_ = 2.617, *p* = 0.106). The *MR* haplotype was not significantly associated with sex or age (both *p* ≥ 0.827).

**Table 1.** Haplotypes in Experiments I and II

		*MR*-2G/C		*MR*-I180V	
rs1512344	rs2070950	**rs2070951**	rs4835519	**rs5522**	rs17658048
**C**	**C**	**C**	**T**	**A**	**G**
**T**	**G**	**G**	**C**	**A**	**A**
**T**	**G**	**G**	**T**	**G**	**G**
**T**	**G**	**G**	**T**	**A**	**G**
**C**	**G**	**G**	**T**	**A**	**G**

Combinations of 6 *MR* SNP alleles as detected by the haplotype analyses in Experiments I and II.

**Table 2.** Haplotype distribution in Experiments I and II

	Experiment I		Experiment II	
Haplotype	Allele frequency	%	Allele frequency	%
**CCCTAG**	250	49.6	125	48.2
**TGGCAA**	173	34.3	89	34.8
**TGGTGG**	58	11.5	29	11.3
**TGGTAG**	18	3.6	10	3.9
**CGGTAG**	4	0.8	-	-
**Unknown**	1	0.2	3	1.2

Allele frequencies and percentage of the haplotypes detected in Experiments I and II. **Unknown** represents participants not carrying the **CCCTAG** haplotype but who could not be assigned to any of the other haplotypes.

#### Successful stress induction by the TSST

**Figure 2.**
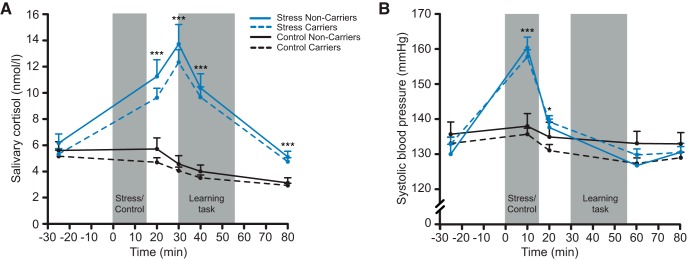
Physiological data of Experiment I. Independent of the *MR* haplotype and compared to a nonstressful control manipulation, exposure to the TSST led to significant increases in (***A***) salivary cortisol concentrations and (***B***) systolic blood pressure. ***p < 0.001, *p < 0.05, error bars represent SEM.

Significant changes in subjective mood, blood pressure and concentrations of the glucocorticoid stress hormone cortisol verified the successful stress induction by the TSST (Table 3). Compared to the control procedure, exposure to the TSST was rated as significantly more difficult, unpleasant and stressful (all *F*_(1248)_ ≥ 165.821, all *p* < 0.001). Moreover, the TSST but not the control manipulation, resulted in increases of depressed mood and restlessness (time × treatment: both *F*_(2246)_ ≥ 37.536, both *p* < 0.001); all participants, irrespective of experimental group, became increasingly tired throughout the experiment (time × treatment: *F*_(1.8440.4)_ = 129.645, *p* < 0.001). In addition, exposure to the TSST led to significant autonomic activation, reflected by increases in systolic (Fig. 2*A*) and diastolic blood pressure (time × treatment: both *F* ≥ 51.146, both *p* < 0.001). Finally, we obtained a significant increase in cortisol concentrations following the stress but not the control manipulation (time × treatment: *F*_(2493.1)_ = 50.777, *p* < 0.001). As shown in Figure 2*B*, peak cortisol levels were reached ∼15 min following the stressor, when behavioral testing started. The *MR* haplotype did not influence measures of blood pressure, cortisol or mood (all *F* ≤ 1.548, all *p* ≥ 0.215).

**Table 3.** Subjective, autonomic, and endocrine stress response in Experiment I

	Control		Stress	
	Carriers	Noncarriers	Carriers	Noncarriers
Subjective assessment				
Stressful	27.62 ± 2.25	28.80 ± 4.01	69.89 ± 2.08	71.11 ± 4.36***
Difficult	28.51 ± 2.31	29.60 ± 4.34	76.00 ± 2.13	73.33 ± 3.40***
Unpleasant	29.50 ± 2.45	27.60 ± 4.25	74.78 ± 2.17	71.39 ± 4.39***
Subjective mood				
Good vs bad mood				
Before treatment	32.65 ± 0.46	32.48 ± 0.77	33.44 ± 0.44	34.14 ± 0.71
1 min after treatment	32.35 ± 0.51	31.76 ± 0.98	26.80 ± 0.69	26.28 ± 1.22***
65 min after treatment	31.19 ± 0.55	31.12 ± 1.02	29.93 ± 0.65	31.61 ± 0.98
Calm vs restless				
Before treatment	30.68 ± 0.59	30.68 ± 0.92	31.23 ± 0.52	31.69 ± 0.96
1 min after treatment	30.19 ± 0.56	29.72 ± 1.10	23.22 ± 0.63	24.06 ± 1.23***
65 min after treatment	31.16 ± 0.57	31.12 ± 1.02	29.60 ± 0.65	32.06 ± 0.95
Tired vs awake				
Before treatment	29.52 ± 0.59	28.00 ± 1.21	30.21 ± 0.68	31.06 ± 1.02
1 min after treatment	29.07 ± 0.65	27.48 ± 1.28	29.36 ± 0.64	30.03 ± 0.99
65 min after treatment	22.85 ± 0.68	23.88 ± 1.39	22.88 ± 0.68	23.89 ± 1.36
Systolic blood pressure (bpm)				
Before treatment	133.02 ± 1.85	135.68 ± 3.50	132.87 ± 1.93	129.99 ± 2.84
During treatment	135.68 ± 1.74	137.98 ± 3.60	157.73 ± 2.05	160.36 ± 3.02***
5 min after treatment	131.05 ± 1.69	134.90 ± 4.20	139.22 ± 1.76	137.59 ± 2.41*
45 min after treatment	127.35 ± 1.59	133.06 ± 3.45	129.72 ± 1.73	126.76 ± 3.23
65 min after treatment	128.95 ± 1.62	132-94 ± 3.23	130.49 ± 1.66	130.56 ± 2.53
Diastolic blood pressure (bpm)				
Before treatment	76.30 ± 0.82	76.88 ± 1.75	76.21 ± 0.92	75.79 ± 1.51
During treatment	80.93 ± 0.79	81.22 ± 2.16	94.62 ± 1.42	94.72 ± 1.92***
5 min after treatment	77.87 ± 0.73	77.20 ± 1.80	81.02 ± 0.95	82.77 ± 1.52**
45 min after treatment	75.23 ± 0.68	75.84 ± 1.65	76.56 ± 0.90	78.11 ± 1.42
65 min after treatment	76.70 ± 0.69	77.26 ± 1.69	76.89 ± 0.91	76.82 ± 1.33
Salivary cortisol (nmol/l)				
Before treatment	5.16 ± 0.46	5.60 ± 0.67	5.32 ± 0.42	6.16 ± 0.70
5 min after treatment	4.69 ± 0.35	5.71 ± 0.85	9.62 ± 0.72	11.25 ± 1.27***
15 min after treatment	4.06 ± 0.27	4.57 ± 0.64	12.33 ± 0.97	13.71 ± 1.50***
25 min after treatment	3.51 ± 0.22	4.00 ± 0.49	9.67 ± 0.79	10.36 ± 1.09***
65 min after treatment	2.93 ± 0.16	3.13 ± 0.39	4.74 ± 0.30	5.11 ± 0.43***

Data represent means ± SEM. bpm, beats per minute. Stress versus control ***p < 0.001, **p < 0.01, *p < 0.05.

#### Carriers of the MR haplotype show enhanced stress-induced shift toward multi-cue strategies

Participants successfully learned the cue-outcome associations, as reflected in a gradual performance improvement from 58 to 74 percent correct responses across PCL trials (*F*_(7.7, 1918.2)_ = 17.730, *p* < 0.001; Fig. 1*C*). In line with previous studies showing that different memory systems may contribute equally well to learning performance (Schwabe and Wolf, 2009, 2012), stress and *MR* haplotype did not influence performance (all *F* ≤ 1.413, *p ≥* 0.240). However, stress tended to change the learning strategies that were used to solve the PCL task: compared to controls, stressed participants tended to engage multi-cue strategies that supposedly depend on the dorsal striatum more often and single-cue strategies that are assumed to depend on the hippocampus less often (χ^2^_(1)_ = 3.200, trend: *p* = 0.074). Most importantly, as shown in Figure 1*D*, stress effects on strategy use differed between *MR* haplotype carriers versus noncarriers. In carriers of this haplotype, stress led to a significant increase in the use of multi-cue strategies from 71-87 percentage and a decrease in the use of single-cue strategies from 29-13 percentage (χ^2^_(1)_ = 7.054, *p* = 0.008, *Cramer’s V* = 0.200). This stress-induced shift in strategy use, was absent in noncarriers of the *MR* haplotype (χ^2^_(1)_ = 0.643, *p* = 0.423, *Cramer’s V* = 0.106). Since previous animal studies suggested that *MR* genotype effects may be sex-dependent (Klok et al., 2011; Vinkers et al., 2015; Hamstra et al., 2017), we performed explorative analyses on our behavioral data, adding sex as another variable in the χ^2^ test. Results indicate that in males, *MR* haplotype carriers use more multi-cue strategies after stress compared to the no-stress control condition (χ^2^_(1)_ = 5.792, *p* = 0.016, *Cramer’s V* = 0.255), whereas in females, there was no significant modulation of strategy use under stress by the *MR* haplotype (χ^2^_(1)_ = 2.091, *p* = 0.148, *Cramer’s V* = 0.154). Similarly, when directly comparing male and female *MR* haplotype carriers in the stress condition, a trend toward a similar effect, namely increased multi-cue strategy use in males, is observed (χ^2^_(1)_ = 2.748, *p* = 0.097, *Cramer’s V* = 0.180), whereas in stressed noncarriers of the *MR* haplotype, we do not detect any gender differences (χ^2^_(1)_ = 0.013, *p* = 0.909, *Cramer’s V* = 0.020). This is in line with evidence showing that female mice with a genetic deletion of forebrain MR continued to use hippocampus-dependent spatial strategies in a maze task despite stress (Ter Horst et al., 2013).

#### Stress and MR haplotype alter electrocortical activity during learning

Our EEG data show that the FRN followed the typical frontocentral distribution and was increased in stressed compared to control participants following negative feedback (*F*_(1223)_ = 9.956, *p* = 0.037; Fig. 1*E*). Since the FRN is particularly important for learning in response to negative feedback (van der Helden et al., 2010), this difference was not observed in response to positive feedback (*F*_(1223)_ = 0.587, *p* = 0.444; feedback × treatment: *F*_(1223)_ = 6.404, *p* = 0.012). Carriers and noncarriers of the *MR* haplotype, neither differed in FRN amplitude (*F*_(1223)_ = 0.254, *p* = 0.615), nor was the stress effect on the FRN modulated by *MR* haplotype (*F*_(1223)_ = 0.646, *p* = 0.422).

In contrast to the FRN, the P3a was reduced in carriers compared to noncarriers of the *MR* haplotype (*F*_(1224)_ = 5.331, *p* = 0.022; Fig. 1*F*), but was not affected by stress (both *F* ≤ 0.043, *p ≥* 0.837), whereas the P3b was neither influenced by the stress manipulation nor by the *MR* haplotype (all *F* ≤ 0.044, *p* ≥ 0.834). Visual inspection of the EEG time course suggested that group differences already developed earlier. Indeed, explorative analyses showed that at frontocentral electrodes the P2 (mean activity 135-235 ms) and the N2 (mean activity 185-285 ms), two early attentional components, were reduced in *MR* haplotype carriers (both *F* ≥ 5.328, *p* ≤ 0.022). Additionally, the P2 tended to be reduced in stressed participants (*F*_(1224)_ = 3.837, *p* = 0.051).

### Experiment II: Neural signature of *MR* haplotype modulation of stress-induced changes in multiple memory systems

Our first experiment showed that the *MR* haplotype modulates the influence of stress on the engagement of different learning strategies in a PCL task. We found evidence that stress led to an increase in FRN amplitude, presumably indicative of increased striatal feedback processing, and that the *MR* haplotype was associated with an increase in early P3a amplitude, which likely reflects cognitive mechanisms that facilitate attention. However, how exactly the *MR* haplotype modulated the stress effect on strategy use on a neural level, remained unclear. Therefore, we ran a second experiment, in which we used fMRI to unravel the neural underpinnings of the modulatory effect of the *MR* haplotype on stress-induced changes in multiple memory systems.

#### MR haplotype analysis

**Figure 3.**
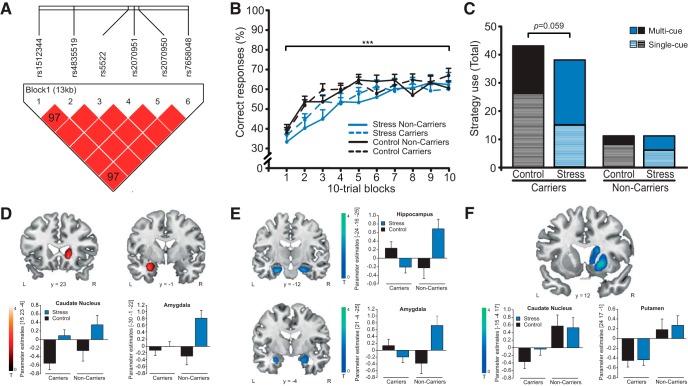
Haplotype analysis, behavioral data and stress and *MR* haplotype effects on brain activity in Experiment II. ***A***, Haplotype block covering 13 kb on the *MR* gene. Homo- and heterozygous *MR* haplotype carriers (*MR*-2G/C **C** [rs2070951], *MR*-I180V **A** [rs5522]) were tested against all other haplotypes. ***B***, Classification learning performance increased across trials but was unaffected by stress or the *MR* haplotype. ***C***, However, stress increased the use of multi-cue strategies, thought to rely on the dorsal striatum, and decreased the use of single-cue strategies, assumed to be supported by the hippocampus. This bias toward enhanced dorsal striatal processing was only observed in stressed *MR* haplotype carriers. Stress increased activation of the caudate nucleus (*p*_FWE_ = 0.035) and the amygdala (*p*_FWE_ = 0.030; ***D***), whereas *MR* haplotype carriers showed enhanced bilateral amygdala activation under stress (both *p*_FWE_ ≤ 0.067) and reduced bilateral hippocampus (both *p*_FWE_ ≤ 0.047; ***E***) and overall reduced activation of the caudate nucleus (*p*_FWE_ = 0.032) and putamen (*p*_FWE_ = 0.006; ***F***). Activations are superimposed on coronal sections of a T1-weighted template image and represented in red. Activation that is reduced in *MR* haplotype carriers is shown in blue. L corresponds to the left, R to the right side of the brain, and error bars represent SEM. ***p < 0.001.

In line with Experiment I, haplotype analyses revealed significantly strong linkage between six *MR* SNPs (rs1512344, rs2070950, **rs2070951**, rs4835519, **rs5522**, rs7658048; Fig. 3*A*). *MR* haplotype details and allele frequencies can be found in Tables 1, 2. This second experiment included 98 carriers of the CC**C**T**A**G haplotype (= *MR* haplotype) that had also been identified in Experiment I (71 participants with one allele and 27 participants with two alleles). Again, the same four other haplotypes were identified and three participants carried unknown haplotypes (Tables 1, 2). In total, 30 participants did not carry the *MR* haplotype. Frequencies of the *MR* SNPs were in accordance with frequencies for Europeans as reported by the NCBI. Frequencies were in Hardy-Weinberg equilibrium (all *p* ≥ 0.11) and genotype was not significantly associated with sex or age (both *F* ≤ 0.376, *p* ≥ 0.540). Carriers and noncarriers of the *MR* haplotype were equally distributed in the stress (50 carriers, 15 noncarriers) and control group (48 carriers, 15 noncarriers; χ^2^_(1)_ = 0.010, *p* = 0.922).

#### Successful stress induction by the TSST

**Figure 4.**
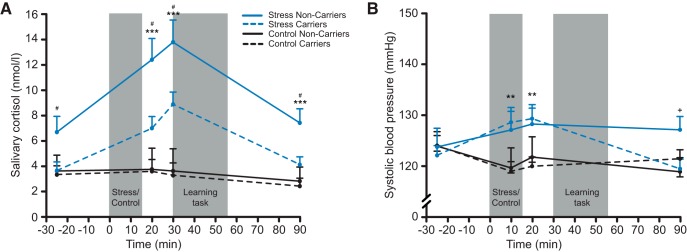
Physiological data of Experiment II. ***A***, Salivary cortisol concentrations were increased in response to the TSST but were generally diminished in *MR* haplotype carriers. ***B***, Similarly, stress exposure led to significant increases in systolic blood pressure and carriers of the *MR* haplotype show increased levels ∼75 min following the stressor. ***p < 0.001, **p < 0.01, *p < 0.05 indicate significant differences between stress and control group; #*p* < 0.05 indicates significant differences between *MR* haplotype carriers and no-carriers; +*p* < 0.05 indicates differences between *MR* haplotype carriers and no-carriers under stress, error bars represent SEM.

As in Experiment I, exposure to the TSST was rated as significantly more difficult, unpleasant and stressful compared to the control manipulation (all *F*_(1124)_ ≥ 46.367, all *p* < 0.001*)* and participants’ mood decreased only following the TSST (all *F*_(2, 246)_ ≥ 12.258, all *p* < 0.001; Table 4). Again, participants became overall increasingly tired throughout the experiment (*F*_(2246)_ = 101.880, *p* < 0.001). Moreover, systolic and diastolic blood pressure as well as salivary cortisol increased following the TSST but not after the control manipulation (all *F* ≥ 10.472, all *p* < 0.001; Fig. 4), with cortisol reaching peak levels shortly before PCL in the MRI scanner.

**Table 4.** Subjective, autonomic, and endocrine stress response in Experiment II

	Control		Stress	
	Carriers	Noncarriers	Carriers	Noncarriers
Subjective assessment				
Stressful	29.17 ± 2.96	30.67 ± 6.28	65.80 ± 2.73	66.00 ± 5.24***
Difficult	26.67 ± 3.01	25.33 ± 6.61	71.80 ± 2.63	67.33 ± 6.93***
Unpleasant	34.38 ± 3.55	38.67 ± 7.61	66.40 ± 3.33	76.00 ± 5.42***
Subjective mood				
Good vs bad mood				
Before treatment	34.65 ± 0.62	33.93 ± 1.09	34.84 ± 0.49	33.07 ± 1.37
1 min after treatment	34.13 ± 0.59	33.27 ± 1.24	28.31 ± 0.92	28.53 ± 1.73***
75 min after treatment	33.21 ± 0.68	31.07 ± 1.44	31.80 ± 0.70	29.27 ± 1.74
Calm vs restless				
Before treatment	31.83 ± 0.79	33.20 ± 0.91	32.26 ± 0.72	29.87 ± 1.33
1 min after treatment	30.63 ± 0.81	30.33 ± 1.67	24.61 ± 0.93	23.53 ± 1.60***
75 min after treatment	31.63 ± 0.77	32.53 ± 1.33	32.38 ± 0.72	30.73 ± 0.91
Tired vs awake				
Before treatment	31.17 ± 0.79	27.67 ± 1.56	31.14 ± 0.65	28.13 ± 1.37
1 min after treatment	30.46 ± 0.85	27.20 ± 1.58	29.61 ± 0.69	25.80 ± 1.62
75 min after treatment	24.65 ± 0.78	19.67 ± 1.41	22.88 ± 0.86	20.47 ± 1.17
Overall tired vs awake	31.36 ± 0.65	32.02 ± 1.15	29.73 ± 0.64	28.04 ± 1.15^##^
Systolic blood pressure (bpm)				
Before treatment	124.09 ± 1.97	124.00 ± 2.84	122.19 ± 1.90	123.83 ± 3.67
During treatment	119.05 ± 1.89	119.73 ± 3.92	128.68 ± 2.14	127.17 ± 4.42**
5 min after treatment	120.04 ± 1.83	121.83 ± 3.99	129.39 ± 2.06	128.33 ± 3.81**
75 min after treatment	121.52 ± 1.77	118.97 ± 2.76	119.55 ± 1.88	127.20 ± 2.61^+^
Diastolic blood pressure (bpm)				
Before treatment	83.25 ± 1.34	82.07 ± 2.53	81.21 ± 0.97	84.90 ± 2.79
During treatment	83.24 ± 1.17	82.50 ± 2.26	89.59 ± 1.49	95.03 ± 3.58***
5 min after treatment	84.07 ± 1.26	83.53 ± 1.83	88.05 ± 1.26	90.47 ± 3.08**
75 min after treatment	84.35 ± 1.42	83.03 ± 1.78	82.90 ± 1.13	86.83 ± 2.51
Salivary cortisol (nmol/l)				
Before treatment	3.37 ± 0.69	3.66 ± 1.24	3.68 ± 0.68	6.71 ± 1.242
5 min after treatment	3.62 ± 0.93	3.78 ± 1.67	7.03 ± 0.91	12.43 ± 1.67***
15 min after treatment	3.31 ± 0.98	3.66 ± 1.75	8.91 ± 0.96	13.80 ± 1.75***
75 min after treatment	2.46 ± 0.62	2.85 ± 1.12	4.16 ± 0.61	7.44 ± 1.12***
Overall salivary cortisol (nmol/l)	3.19 ± 0.75	3.48 ± 1.34	5.94 ± 0.73	10.10 ± 1.34^#^

Data represent means ± SEM. bpm, beats per minute. Time × treatment (stress vs control) interaction ***p < 0.001 and **p < 0.01. *MR* haplotype (carriers vs noncarriers) main effect ##*p* < 0.01 and #*p* < 0.05. Time × treatment × *MR* haplotype interaction +*p* < 0.05.

Although the subjective response to the stressor was not affected by the *MR* haplotype (all *F*_(1123)_ ≤ 1.814, *p* ≥ 0.165), carriers of the *MR* haplotype felt more awake throughout the experiment (*F*_(1123)_ = 14.359, *p* < 0.001). Moreover, the *MR* haplotype affected blood pressure and cortisol levels. Approximately 75 min after the TSST, the *MR* haplotype was associated with decreased systolic blood pressure levels (time × treatment × *MR* haplotype: *F*_(2121)_ = 4.079, *p* < 0.001; *post hoc* comparison of time point of measurement: *p* = 0.044). Diastolic blood pressure remained unaffected by the *MR* haplotype (time × *MR* haplotype: *F*_(2.5311)_ = 0.330, *p* = 0.769; main effect *MR* haplotype: *F*_(1123)_ = 0.777, *p* = 0.380). Salivary cortisol was reduced in carriers compared to noncarriers of the *MR* haplotype irrespective of the experimental condition (*F*_(1124)_ = 4.224, *p* = 0.042; Fig. 4*A*). Furthermore, although not statistically significant, carriers of the *MR* haplotype tended to show an attenuated cortisol response to the TSST (*p* = 0.077).

#### MR haplotype is associated with enhanced stress-induced bias toward multi-cue strategies

Participants gradually learned to correctly classify the cues and correct responses increased from 37 to 62% across PCL trials (*F*_(6.7,828.5)_ = 20.901, *p* < 0.001; Fig. 3*B*). Stress and *MR* haplotype had no effect on task performance (all *F* ≤ 2.916, all *p* ≥ 0.90). Corroborating the behavioral findings of Experiment I, stress led, compared to the control manipulation, to more multi-cue and less single-cue learning (χ^2^_(1)_ = 4.173, *p* = 0.041). This stress-induced shift in learning strategy was modulated by the *MR* haplotype. As shown in Figure 3*C*, only *MR* haplotype carriers tended to shift toward multi-cue strategies after stress (χ^2^_(1)_ = 3.556, *p* = 0.059, *Cramer’s V* = 0.210), whereas there was no such effect in noncarriers of this haplotype (χ^2^_(1)_ = 0.786, *p* = 0.375, *Cramer’s V* = 0.189). The effect sizes for the modulatory effect of the MR haplotype on the stress-induced bias toward multi-cue strategies were comparable between Experiment I (*Cramer’s V* = 0.2) and Experiment II (*Cramer’s V* = 0.21), indicating that the trend-level significance of the MR modulation in this fMRI experiment was most likely due to lower statistical power. Although in our first experiment explorative analyses revealed that the modulatory effects of the *MR* haplotype on strategy use under stress may be sex dependent, the smaller sample size of this fMRI experiment did not allow for such analyses.

#### Neural underpinnings of the MR haplotype-dependent modulation of multiple memory systems after stress

Corroborating previous studies (Poldrack et al., 2001; Foerde et al., 2006; Schwabe and Wolf, 2012; Schwabe et al., 2013), PCL (vs visuomotor control trials) led to bilateral activation of the caudate nucleus, putamen and hippocampus (all *p*_FWE_ ≤ 0.031). In addition, PCL activated regions such as the cingulate and paracingulate cortex, orbitofrontal cortex, insular cortex and precuneus (all *p*_FWE_ < 0.001; Table 5). Stress led to a significant increase in caudate activation during learning (*t* =3.21, *p*_FWE_ = 0.035, *k*: 34; Fig. 3*D*). Whereas this stress-induced increase in dorsal striatal activity was not modulated by the *MR* haplotype, the *MR* haplotype affected activation in the hippocampus under stress, in that stressed *MR* haplotype carriers showed significantly reduced bilateral hippocampal activation (right: *t* = 3.15, *p*_FWE_ = 0.047, *k*: 32; left: *t* = 3.48, *p*_FWE_ = 0.019, *k*: 38; Fig. 3*E*). In addition, stress increased amygdala activity (*t* = 3.01, *p*_FWE_ = 0.030, *k*: 20) and this stress-induced increase was modulated by the *MR* haplotype, with stressed participants not carrying the *MR* haplotype showing greater bilateral amygdala activation (right: *t* = 2.98, *p*_FWE_ = 0.038, *k*: 20; left: *t* = 2.67, *p*_FWE_ = 0.067, *k*: 21; Fig. 3*E*). Irrespective of stress manipulation, the *MR* haplotype was associated with reduced activation of the caudate nucleus (*t* = 3.25, *p*_FWE_ = 0.032, *k*: 69) and the putamen (*t* = 3.93, *p*_FWE_ = 0.006, *k*: 56; Fig. 3*F*).

**Table 5.** Significantly activated cluster peak voxels and *T* values during PCL

	MNI coordinates (mm)					
PCL > control	Cluster size	*x*	*y*	*z*	*T*_max_	*p*_corr_
L supplementary motor area	866	0	23	47	19.51	<0.001
L insula left; L caudate; R caudate	3.083	-30	20	-4	18.32	<0.001
R insula; R inferior frontal gyrus triangular; R precentral gyrus	900	33	20	-4	17.11	<0.001
L inferior parietal sulcus; R angular gyrus; L precuneus	2.337	-33	-58	44	15.02	<0.001
R middle occipital gyrus; R cuneus; R fusiform gyrus	879	33	-88	-1	12.61	<0.001
L middle occipital gyrus left; inferior temporal gyrus	512	-15	-103	2	9.03	<0.001
Middle cingulate cortex	101	-3	-28	29	8.54	<0.001
L middle frontal gyrus	58	-30	5	56	6.68	<0.001
R anterior orbitofrontal cortex	20	27	38	-22	6.29	<0.001
Cerebellar crus	11	-36	-61	-28	5.83	0.001
Calcarine cortex	9	3	-88	-7	5.69	0.001
R anterior orbitofrontal cortex	5	48	47	-16	5.66	0.001
R middle frontal gyrus; superior frontal gyrus	42	33	53	2	5.22	0.008
L anterior cingulate cortex	5	-6	-1	29	5.18	0.010
L hippocampus CA	7	-18	-37	5	3.33	0.031*
R hippocampus CA	23	18	-34	2	6.89	<0.001*
L hippocampus DG	5	-21	-37	2	3.97	0.001*
L hippocampus DG	5	21	-34	2	6.89	<0.001*
L hippocampus	5	-21	-28	-10	3.91	0.005*
L caudate nucleus	106	-9	11	-1	15.55	<0.001*
R caudate nucleus	107	9	11	2	14.87	<0.001*
L putamen	38	-15	8	-4	10.96	<0.001*
R putamen	36	18	11	-1	7.32	<0.001*

Table shows local maxima of functional voxels (normalized voxel size = 3 × 3 × 3 mm³). MNI, Montreal Neurologic Institute; corr, corrected. All labels are taken from the Automatic Anatomic Labeling (ALL) atlas. The significance threshold was set to *p* < 0.05 (FWE corrected). *, small volume corrected; all other activations are sig. at the whole-brain level.

#### MR haplotype modulates stress-induced changes in amygdala connectivity with the dorsal striatum and the hippocampus

Because previous evidence suggested that the amygdala may orchestrate the engagement of multiple memory systems under stress (Vogel et al., 2016), we analyzed functional connectivity of the amygdala with the hippocampus, caudate nucleus and putamen. In line with a stress-induced modulation of multiple memory systems at the expense of the hippocampus-dependent system, stressed participants showed decreased amygdala connectivity with the cornu ammonis subregion of the hippocampus (*t* = 3.23, *p*_FWE_ = 0.043, *k*: 318) as well as a trend toward decreased amygdala-entorhinal cortex coupling (*t* = 2.95, *p*_FWE_ = 0.061, *k*: 10; Fig. 5*A*). Critically, the *MR* haplotype modulated the connectivity of the amygdala with structures of the cognitive and habitual systems under stress (treatment × *MR* haplotype interactions all *t* ≥ 2.83, *p*_FWE_ ≤ 0.065, all *k* ≥ 24). In the stress condition, amygdala connectivity with the anterior parahippocampal region was reduced in carriers of the *MR* haplotype (right: *t* = 3.01, *p*_FWE_ = 0.032, *k*: 24; left: *t* = 2.91, *p*_FWE_ = 0.063, *k*: 29; Fig. 5*B*). Conversely, in the control condition, amygdala connectivity with the caudate nucleus was increased in *MR* haplotype carriers relative to noncarriers (*t* = 3.58, *p*_FWE_ = 0.018, *k*: 44; Fig. 5*B*).

**Figure 5.**
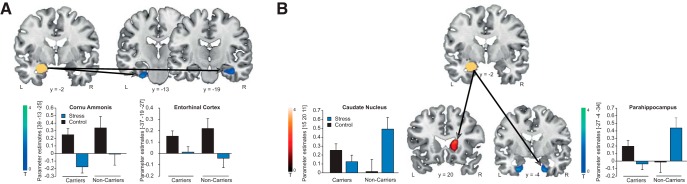
Stress and *MR* haplotype effects on brain connectivity in Experiment II. ***A***, Stress resulted in decreased amygdala-hippocampus connectivity (cornu ammonis: *p*_FWE_ = 0.043, enthorhinal cortex: *p*_FWE_ = 0.061). *MR* haplotype carriers showed reduced amygdala-anterior parahippocampus connectivity under stress (both *p*_FWE_ ≤ 0.063; ***B***), whereas under control conditions, *MR* haplotype carriers showed increased amygdala-caudate nucleus coupling (*p*_FWE_ = 0.018; ***C***). Activations are superimposed on coronal sections of a T1-weighted template image and represented in red when greater blue when reduced in *MR* haplotype carriers, the anatomic mask is indicated in yellow. L corresponds to the left, R to the right side of the brain, and error bars represent SEM.

#### Role of altered cortisol levels in the MR haplotype-dependent modulation of multiple memory systems under stress

Because carriers of the *MR* haplotype showed overall lower cortisol concentrations and tended to have a reduced cortisol response to the TSST, we tested whether the behavioral and neuronal effects of the *MR* haplotype were mediated by altered cortisol levels or whether these effects occurred independently of changes in cortisol responses. Results of mediation and moderation analyses using the PROCESS plugin for SPSS (Hayes, 2013) showed that cortisol (expressed as area under the curve with respect to ground) neither moderated nor mediated the stress-induced increase in dorsal striatum-dependent, multi-cue strategies in *MR* haplotype carriers (moderation *p* = 0.930, mediation *p* = 0.562). Similarly, our imaging data remained largely unchanged after including cortisol as a covariate. Activation of the hippocampus and the amygdala was still reduced in stressed *MR* haplotype carriers (hippocampus: right: *t* = 3.16, *p*_FWE_ = 0.046, *k*: 30; left: *t* = 3.46, *p*_FWE_ = 0.020, *k*: 39) and ireespective of the stress manipulation, putamen activity was still reduced in *MR* haplotype carriers (*t* = 3.44, *p*_FWE_ = 0.025, *k*: 38), whereas the significant caudate nucleus activation became a trend (*t* = 2.91, *p*_FWE_ = 0.075, *k*: 21). The reduced amygdala-anterior parahippocampus connectivity in stressed *MR* haplotype carriers dropped to trend level (right: *t* = 2.66, *p*_FWE_ = 0.095, *k*: 9; left: *t* = 2.55, *p*_FWE_ = 0.109, *k*: 6). However, in the control condition, increased amygdala-caudate nucleus connectivity in carriers of the *MR* haplotype remained significant (*t* = 3.13, *p*_FWE_ = 0.040, *k*: 28).

## Discussion

It is increasingly acknowledged that stress, whether acute or chronic, promotes a shift from more complex, cognitive toward rather simple but rigid forms of learning and memory (Schwabe et al., 2008; Dias-Ferreira et al., 2009; Packard and Goodman, 2012; Soares et al., 2012; Schwabe and Wolf, 2012; Schwabe and Wolf, 2013; Vogel et al., 2016; Wirz et al., 2018). Although this shift might contribute to stress-related psychopathology (Packard, 2009; Schwabe et al., 2011; de Quervain et al., 2017), not all individuals are equally susceptible to this stress-induced bias. Here, we showed in two independent experiments that a haplotype containing one or two copies of the alleles of two functional *MR* SNPs previously associated with enhanced MR expression (*MR*-2G/C **C**, *MR*-I180V **A**; DeRijk and De Kloet, 2008) facilitates the stress-induced shift from hippocampus-dependent toward dorsal striatum-dependent memory. In fact, the stress-induced shift toward dorsal striatal processing was solely observed in carriers of this haplotype. This modulation of the stress-induced shift toward habit memory by the *MR* haplotype was accompanied by specific changes in memory networks, indicating that the influence of this haplotype was mainly linked to impaired hippocampal processing and reduced amygdala-hippocampus cross talk under stress.

Since the discovery of membrane-bound MRs (Karst et al., 2005), several studies have demonstrated a role of rapid, nongenomic MR signaling in cognition. For instance, MR antagonists have been shown to impair selective attention and working memory performance and to enhance long-term memory (Otte et al., 2007; Cornelisse et al., 2011), whereas MR agonists have been shown to improve verbal memory and executive function in depressed patients (Otte et al., 2015) and to be associated with risky decision making (Deuter et al., 2017). Moreover, converging lines of evidence from rodent and human experiments pointed to a critical role of the MR in the engagement of multiple memory systems under stress. Specifically, pharmacological blockade of the MR prevented the shift from hippocampal toward dorsal striatal learning strategies as well as stress-induced alterations in amygdala connectivity with the hippocampus and dorsal striatum, respectively (Schwabe and Wolf, 2012; Schwabe et al., 2013; Vogel et al., 2015). Here we show that individual differences in the *MR* gene, involving two SNPs with known functionality, modulate the shift in the engagement of multiple memory systems under stress and may therefore explain at least part of the individual variability in this shift. Our finding that an *MR* haplotype associated with increased MR expression and transactivational activity is associated with increased probability of engaging the dorsal striatum-dependent memory system under stress is in line with the previous pharmacological data (Schwabe et al., 2010b, 2013) and underlines the critical involvement of the MR in stress effects on the engagement of multiple memory systems. Interestingly and further in line with previous evidence for the stress-induced shift in the engagement of multiple memory systems (Schwabe and Wolf, 2009, 2012), neither stress nor the MR haplotype affected actual task performance. This underlines that both systems can support performance. The impact of the engaged system, however, may be seen when the learning environment changes and the flexibility of learned is probed (Schwabe and Wolf, 2013; Quaedflieg and Schwabe, 2017).

Using EEG and fMRI, we investigated the neural underpinnings of the role of the *MR* haplotype in the stress-induced modulation of multiple memory systems. Our EEG data showed that stress was overall associated with a larger FRN, suggesting increased striatal processing (Nieuwenhuis et al., 2005; Foti et al., 2011), in line with the assumed bias toward dorsal striatal learning after stress. The *MR* haplotype was, irrespective of stress, associated with a reduced P3a, an ERP component related to attentional and memory processes as well as hippocampal functioning (Knight, 1996; Polich, 2007). Additionally, already earlier components (P2, N2) were reduced in *MR* haplotype carriers, suggesting that early attentional processes are affected by differences in MR functionality. Corroborating an influence of the *MR* haplotype on hippocampal processing, our fMRI data revealed that stress reduced hippocampal activity, particularly in carriers of the *MR* haplotype. Together these data suggest that the *MR* haplotype is generally linked to reduced processing in cognitive areas such as the hippocampus, which may render these areas in *MR* haplotype carriers particularly vulnerable to the impact of stress. Reduced hippocampal involvement in learning may allow the dorsal striatum to dominate learning under stress. Indeed, in contrast to the hippocampus and in line with our EEG findings, stress led to increased striatal activity during learning, irrespective of genotype. Whereas these *MR* haplotype- and stress-related changes in hippocampal and dorsal striatal activity fit very well with the existing literature, the finding that the *MR* haplotype was associated with attenuated amygdala activation after stress and, under no-stress conditions, with reduced caudate and putamen activity was less expected. Although these latter results clearly require further investigation, the reduced activations might reflect more efficient processing in *MR* haplotype carriers (Rypma et al., 2006), enabling them to shift more easily to the dorsal striatal system after stress.

In addition to these changes in single brain areas, the *MR* haplotype modulated stress-induced alterations in connectivity of the amygdala with multiple memory systems. Previous findings showed that stress increases amygdala connectivity with the dorsal striatum but reduces amygdala connectivity with the hippocampus and that these opposite changes in amygdala connectivity are abolished by an MR antagonist (Schwabe et al., 2013). In line with these findings, we obtained reduced amygdala-hippocampus connectivity under stress and the stress-induced decrease in amygdala cross talk with medial temporal cortices adjacent to the hippocampus (in particular, the parahippocampal cortex) was present only in carriers of the *MR* haplotype.

Beyond its role in cognition, the MR has been associated with negative feedback control of the HPA axis. Accordingly, pharmacological manipulations of MR functioning typically result in altered cortisol levels (Schwabe et al., 2010b; Cornelisse et al., 2011; Schwabe et al., 2013; Otte et al., 2015) and two *MR* polymorphisms have been linked to altered cortisol responses to stress (DeRijk et al., 2006; DeRijk, 2009). Since we obtained reduced cortisol levels in carriers of the MR haplotype, as one would expect in carriers of MR variants associated with enhanced functioning, only in Experiment II but not in Experiment I, our present data remain inconclusive with respect to the role of the MR haplotype in the modulation of the HPA axis. Even more important, however, is the question whether effects of the *MR* haplotype on the engagement of multiple memory systems are mainly related to altered cortisol responses. The fact that we observed an influence of the *MR* haplotype on the engagement of multiple memory systems under stress in both experiments, while its impact on cortisol concentrations was only present in one of the experiments, renders a mere dependency on different cortisol levels unlikely. Moreover, we did not find any effects of cortisol when directly testing for mediation or moderation effects and our neuroimaging results remained largely unchanged when cortisol was added as a covariate. Thus, we argue that the behavioral and neural effects of the *MR* haplotype are not owing to an altered cortisol response to a stressor, but most likely to increased efficiency in how cortisol binding to MRs induces a shift toward the dorsal striatum, i.e. in how cortisol acting through the MR can translate into behavioral changes. Determining how exactly a genetic variation in the *MR* gene translates into a more pronounced bias toward habit learning under stress remains a challenge for future molecular studies. Importantly, future studies will need to investigate additive gene dose-dependent effects of the *MR* haplotype (Hamstra et al., 2017) as well as sex-dependent effects (Vinkers et al., 2015). In particular, explorative analyses of the behavioral data of our first experiment lent some support for sex-dependent differences in the interactive influence of stress and *MR* genotype on the engagement of multiple learning strategies. Specifically, only male *MR* haplotype carriers showed enhanced use of multi-cue strategies after stress, which is in line with previous evidence in rodents (Ter Horst et al., 2013). As sex differences were not the focus of this study, the present analyses of potential sex effects are rather preliminary. Given the potential relevance of such effects in the face of different prevalences of stress-related mental disorders in men and women (Bangasser and Valentino, 2014), determining whether there are significant differences in stress × *MR* genotype interactions on the use of multiple memory systems between men and women is an important challenge for future studies. Similarly, and in line with the finding that also genetic differences in the noradrenergic system modulate stress effects on multiple memory systems (Wirz et al., 2017), it will be important for future research to investigate interactive effects of several genes, which will allow pooling of the relative small effects of individual polymorphisms.

Because of differences in the temporal resolution of the EEG and fMRI measurements, feedback timing varied between the two experiments. Importantly, whereas the striatum is highly important for immediate feedback processing, the engagement of the hippocampus increases when feedback is delayed (Foerde and Shohamy, 2011). In line with this idea, there was overall a higher percentage of single-cue strategies in our EEG experiment, in which feedback followed shortly after the response, whereas in our fMRI study, due to the slow BOLD response, feedback was delayed, leading to a generally stronger engagement of the dorsal striatal memory system. Critically, however, stress increased multi-cue learning and the *MR* haplotype modulated this effect, irrespective of these timing differences and the general differences in the distribution of the strategies between the experiments.

To conclude, we showed in two independent experiments that genetic variations in several *MR* SNPs synergistically modulate the stress-induced bias toward dorsal striatal memory and thus explain at least part of the individual variance in this bias. Although the stress-induced shift from hippocampus-dependent cognitive toward dorsal striatum-dependent habit memory may impair memory flexibility (Seehagen et al., 2015), it is thought to be generally beneficial for coping with a stressor (Vogel et al., 2016). The ability to shift flexibly between these systems may have important implications for stress-related mental disorders such as PTSD, for which glucocorticoid-based therapeutic approaches have been proposed (de Quervain et al., 2017). In order for such interventions to be successful, personalized treatment strategies taking individual vulnerability to stress-induced changes in cognition into account are crucial. Our data suggest that, in addition to genetic variations of glucocorticoid and adrenergic receptors (de Quervain et al., 2017; Wirz et al., 2017), genetic variations of the MR may be very important in this respect.

## Synthesis

Reviewing Editor: Carmen Sandi, Swiss Federal Institute of Technology Decisions are customarily a result of the Reviewing Editor and the peer reviewers coming together and discussing their recommendations until a consensus is reached. When revisions are invited, a fact-based synthesis statement explaining their decision and outlining what is needed to prepare a revision will be listed below. Note: If this manuscript was transferred from JNeurosci and a decision was made to accept the manuscript without peer review, a brief statement to this effect will instead be what is listed below.

A mineralocorticoid receptor (MR)-dependent switch in learning strategy was discovered in pioneering animal research a few years ago and later extended to humans. Here, Wirz, Schwabe and collaborators investigated the effects of mineralocorticoid receptor (MR) gene variation in mediating stress-induced bias from hippocampus-dependent learning toward dorsal striatum-dependent 'habit' learning. In two experiments, the authors report that genetic variation of the mineralocorticoid receptor is associated with a stress-induced switch in learning strategy. They find that homozygote + heterozygote carriers of the haplotype 2 C/A (rs 2070951 and rs 5522) rather than non-carriers associate with a stress-induced reduction in a hippocampal based strategy in the modified weather prediction task, a probabilistic classification learning task validated previously by the same investigators. This reduction of a hippocampal-based cognitive strategy is supported by EEG data (P3 signal) and in a replicate sample with fMRI. Systolic blood pressure and saliva cortisol concentration were also analyzed in a haplotype dependent fashion. The stressor was the classical Trier Social Stress Test (TSST).

A particular strength of the paper is that it combines state-of-the-art approaches, including neuropsychological testing, neuro-imaging, EEG and TSST response characteristics with MR genetics in humans. This approach allows revealing the complex interaction between corticosteroid genes on the one hand, and neural systems involved in learning and memory (striatum, hippocampus, etc.) on the other.
